# Supplementary material for: Reconstitution of the SARS-CoV-2 ribonucleosome provides insights into genomic RNA packaging and regulation by phosphorylation
Source: J Biol Chem. 2022 Oct 4;298(11):102560. doi: 10.1016/j.jbc.2022.102560 (PMC9529352; doi:10.1016/j.jbc.2022.102560)
Supplement: Supplementary Tables and Figures [file mmc1.pdf]

Supporting Information

Reconstitution of the SARS-CoV-2 ribonucleosome provides insights into genomic RNA packaging and regulation by phosphorylation

Christopher R. Carlson, Armin N. Adly, Maxine Bi, Conor J. Howard, Adam Frost, Yifan Cheng, and David O. Morgan

Figures S1-S4

Tables S1, S2

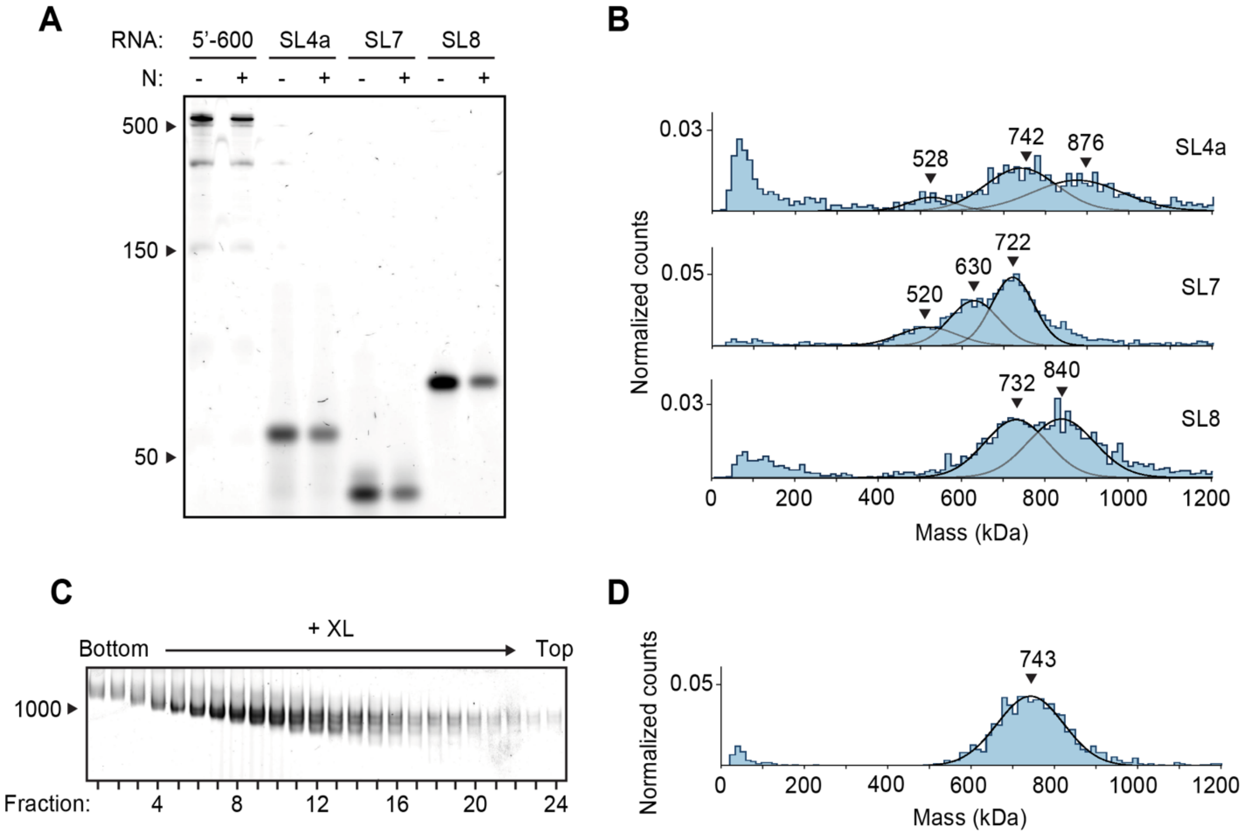

Figure S1. vRNP formation with stem-loop RNAs. *A*, Denaturing gel electrophoresis of N protein mixed with indicated RNAs, from samples shown in Fig. 2*B*. RNA length standards shown on left (nt). *B*, Mass photometry analyses of crosslinked N protein complexes with indicated RNAs. Results with SL8 are reproduced in Fig. 2*D*. Representative of two independent experiments (Table S1). *C*, N protein in complex with SL8 RNA was separated by glycerol gradient centrifugation in the presence of crosslinker (GraFix) and analyzed by native gel electrophoresis. RNA length standard shown on left (nt). *D*, Fractions 7 and 8 of GraFix-purified N-SL8 vRNPs (from *C*) were combined and analyzed by mass photometry. Representative of two independent experiments (Table S1).

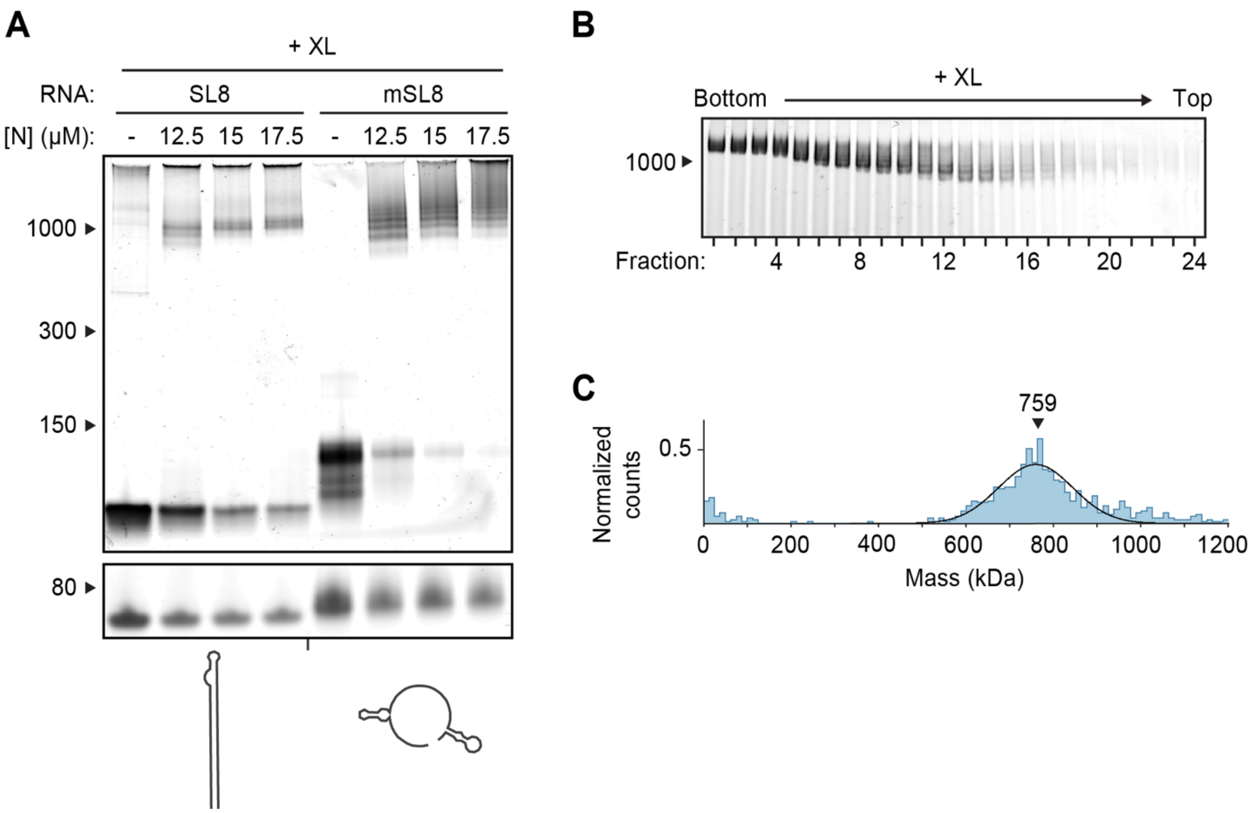

Figure S2. Analysis of vRNP formation with mSL8 RNA. *A*, The indicated concentrations of N protein were combined with SL8 RNA or mutant SL8 RNA (mSL8), crosslinked, and analyzed by native (top) and denaturing (bottom) gel electrophoresis. RNA length standards shown on left (nt). Predicted secondary structures are shown below. See Table S2 for sequences. *B*, N protein in complex with mSL8 RNA was separated by glycerol gradient centrifugation in the presence of crosslinker (GraFix) and analyzed by native gel electrophoresis. RNA length standard shown on left (nt). *C*, Fractions 7 and 8 of GraFix-purified N-mSL8 vRNPs (from *B*) were combined and analyzed by mass photometry. Representative of two independent experiments (Table S1).

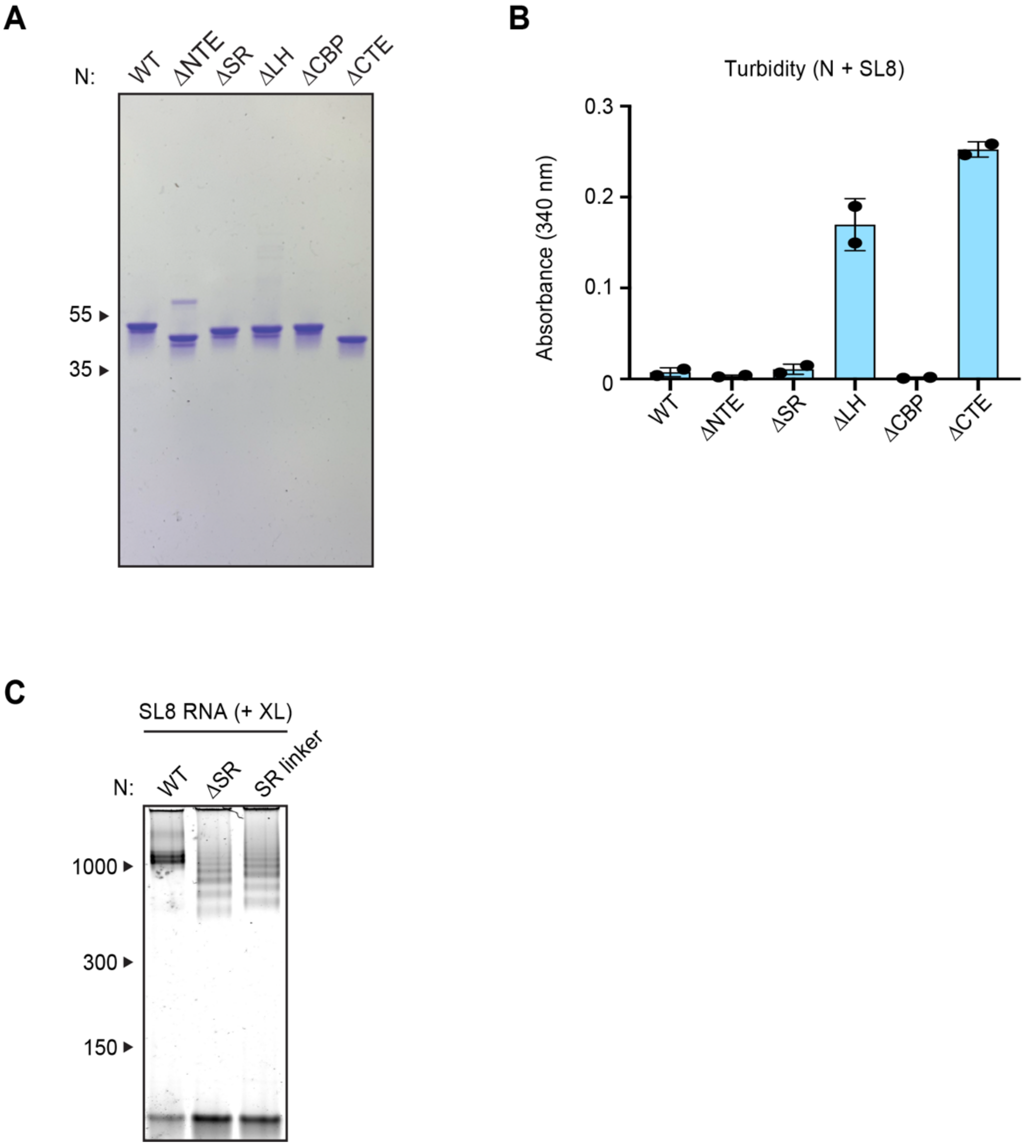

Figure S3. Analysis of N protein mutants. *A*, SDS-PAGE of N protein constructs, stained with Coomassie Blue. Molecular mass markers at left (kDa). *B*, Absorbance at 340 nm was used to quantify the turbidity of wild-type and mutant N proteins mixed with SL8 RNA. All values are normalized to absorbance at 260 nm. Bars indicate means ± SD (n=2). *C*, 16 μM N protein mutants were mixed with 256 ng/μl SL8 RNA, crosslinked, and analyzed by native gel electrophoresis. RNA length standards shown on left (nt). In the SR linker mutant, amino acids 176 to 206 are replaced with the sequence GASGGAGASGGAGASGGAGASGGAGASGGAA.

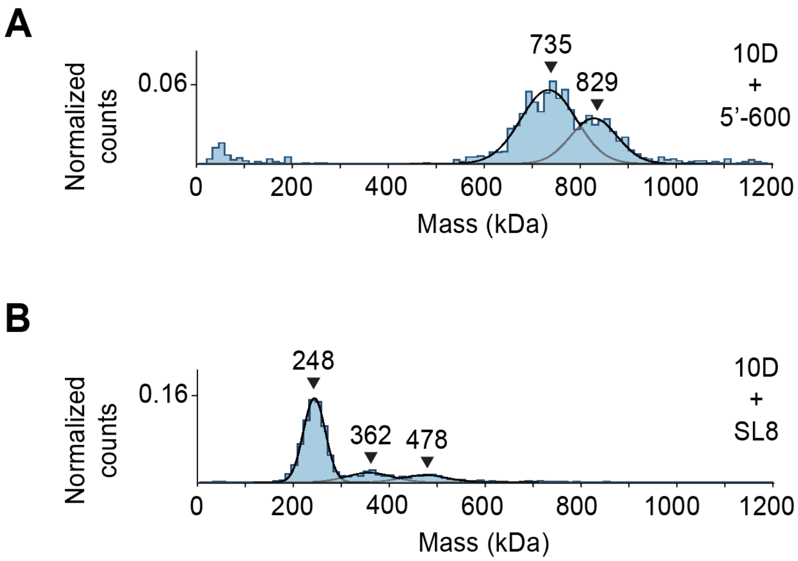

Figure S4. Analysis of complex formation by 10D mutant. Mass photometry analysis of GraFix-purified fractions 7 + 8 of 10D protein in complex with 5'-600 RNA (*A*) and fractions 19 + 20 of 10D protein in complex with SL8 RNA (*B*). Representative of two independent experiments (Table S1).

Table S1. Summary of mass photometry results (kDa).

	Figure 1								
	Figure 1C								
	5'-400			5'-600			5'-800		
peak	rep 1	rep 2	mean ± SD	rep 1	rep 2	mean ± SD	rep 1	rep 2	mean ± SD
1	630	631	630.5 ± 0.7	802	853	827.5 ± 36	756	721	738.5 ± 24.7
2	739	728	733.5 ± 7.8				1418	1613	1515.5 ± 137.9

	Figure 1E					
	-XL			+XL		
peak	rep 1	rep 2	mean ± SD	rep 1	rep 2	mean ± SD
1	95	98	96.5 ± 2.2	724	714	719 ± 7.1
2	202	211	206.5 ± 6.4			
3	761	770	765.5 ± 6.4			

	Figure 2									
	Figure 2D									
	-SL8 / -XL			+SL8 / -XL			+SL8 / +XL (from Fig S1B)			
peak	rep 1	rep 2	mean ± SD	rep 1	rep 2	mean ± SD	rep 1	rep 2	rep 3	mean ± SD
1	96	98	97 ± 1.4	110	105	107.5 ± 3.5	732	728	749	736.5 ± 11.2
2	196	202	199 ± 4.2	232	218	225 ± 9.9	840	829	852	840.3 ± 11.5
3				361	358	359.5 ± 2.1				
4				487	448	467.5 ± 27.6				
5				622	578	600 ± 31.1				
6				755	717	736 ± 26.9				

	Figure 3									
	Figure 3D									
	WT (from Fig S1B)				ΔNTE			ΔSR		
peak	rep 1	rep 2	rep 3	mean ± SD	rep 1	rep 2	mean ± SD	rep 1	rep 2	mean ± SD
1	732	728	749	736.5 ± 11.2	701	702	701.5 ± 1	236	224	230 ± 8.5
2	840	829	852	840.3 ± 11.5	775	804	789.5 ± 20.5	357	324	340.5 ± 23.3
3								472	432	452 ± 28.3
4								589	552	570.5 ± 26.2
5								706	689	697.5 ± 12
6								844	831	837.5 ± 9.2

	Figure 3D (continued)								
	ΔLH			ΔCBP			ΔCTE		
peak	rep 1	rep 2	mean ± SD	rep 1	rep 2	mean ± SD	rep 1	rep 2	mean ± SD
1	100	113	106.5 ± 9.2	92	107	99.5 ± 10.6	n/a	n/a	n/a
2	214	230	222 ± 11.3	200	215	207.5 ± 10.6			
3	335	345	340 ± 7.1	303	332	317.5 ± 20.5			
4				428	436	432 ± 5.7			
5				562	568	565 ± 4.2			
6									

	Figure 4									
	Figure 4D									
	WT (from Fig S1B)				ΔSR (from fig 3)			10D		
peak	rep 1	rep 2	rep 3	mean ± SD	rep 1	rep 2	mean ± SD	rep 1	rep 2	mean ± SD
1	732	728	749	736.5 ± 11.2	236	224	230 ± 8.5	231	230	230.5 ± 0.7
2	840	829	852	840.3 ± 11.5	357	324	340.5 ± 23.3	346	348	347 ± 1.4
3					472	432	452 ± 28.3	456	452	454 ± 2.8
4					589	552	570.5 ± 26.2	569	540	554.5 ± 20.5
5					706	689	697.5 ± 12			
6					844	831	837.5 ± 9.2			

	Figure 5					
	Figure 5D					
	WT (+kinases)			S188A + S206A (+kinases)		
peak	rep 1	rep 2	mean ± SD	rep 1	rep 2	mean ± SD
1	235	207	221 ± 19.8	736	730	733 ± 4.2
2	335	308	321.5 ± 19.1			
3	445	447	446 ± 1.4			

	Figure S1									
	Figure S1B									
	SL4a			SL7			SL8			
peak	rep 1	rep 2	mean ± SD	rep 1	rep 2	mean ± SD	rep 1	rep 2	rep 3	mean ± SD
1	528	501	514.5 ± 19.1	520	484	502 ± 25.5	732	728	749	736.5 ± 11.2
2	742	735	738.5 ± 4.9	630	599	614.5 ± 21.9	840	829	852	840.3 ± 11.5
3	876	875	875.5 ± 0.7	722	703	712.5 ± 13.4				

	Figure S1D		
	SL8 (GRAFIX)		
peak	rep 1	rep 2	mean ± SD
1	743	736	739.5 ± 4.9

	Figure S2		
	Figure S2C		
	WT +mSL8 (GRAFIX)		
peak	rep 1	rep 2	mean ± SD
1	759	773	766 ± 9.9

	Figure S4		
	Figure S4A		
	10D + 5'-600 (GRAFIX)		
peak	rep 1	rep 2	mean ± SD
1	735	725	730 ± 7.1
2	829	817	823 ± 8.5

	Figure S4B		
	10D + SL8 (GRAFIX)		
peak	rep 1	rep 2	mean ± SD
1	248	249	248.5 ± 0.7
2	362	367	364.5 ± 3.5
3	478	487	482.5 ± 6.4

Table S2. RNA sequences used in this study.

5’-400	auuaaagguuuauaccuucccagguaacaaaccaaccaacuuucgaucucuuguagaucuguucucu<br>aaacgaacuuuaaaaucuguguggcugucacucggcugcaugcuuagugcacucacgcaguauaauu<br>aauaacuaauuacugucguugacaggacacgaguaacucgucuaucuucugcaggcugcuuacgguu<br>ucguccguguugcagccgaucaucagcacaucuagguuucguccgggugugaccgaaagguaagaug<br>gagagccuugucccugguuucaacgagaaaacacacguccaacucaguuugccuguuuuacagguuc<br>gcgacgugcucguacguggcuuuggagacuccguggaggaggucuuaucagaggcacgucaacau
5’-600	auuaaagguuuauaccuucccagguaacaaaccaaccaacuuucgaucucuuguagaucuguucucu<br>aaacgaacuuuaaaaucuguguggcugucacucggcugcaugcuuagugcacucacgcaguauaauu<br>aauaacuaauuacugucguugacaggacacgaguaacucgucuaucuucugcaggcugcuuacgguu<br>ucguccguguugcagccgaucaucagcacaucuagguuucguccgggugugaccgaaagguaagaug<br>gagagccuugucccugguuucaacgagaaaacacacguccaacucaguuugccuguuuuacagguuc<br>gcgacgugcucguacguggcuuuggagacuccguggaggaggucuuaucagaggcacgucaacaucu<br>uaaagauggcacuuguggcuuaguagaaguugaaaaaggcguuuugccucaacuugaacagcccuau<br>guguucaucaaacguucggaugcucgaacugcaccucauggucauguuaugguugagcugguagcag<br>aacucgaaggcauucaguacggucguaguggugagacacuugguguccuugucccucaugugg
5’-800	auuaaagguuuauaccuucccagguaacaaaccaaccaacuuucgaucucuuguagaucuguucucu<br>aaacgaacuuuaaaaucuguguggcugucacucggcugcaugcuuagugcacucacgcaguauaauu<br>aauaacuaauuacugucguugacaggacacgaguaacucgucuaucuucugcaggcugcuuacgguu<br>ucguccguguugcagccgaucaucagcacaucuagguuucguccgggugugaccgaaagguaagaug<br>gagagccuugucccugguuucaacgagaaaacacacguccaacucaguuugccuguuuuacagguuc<br>gcgacgugcucguacguggcuuuggagacuccguggaggaggucuuaucagaggcacgucaacaucu<br>uaaagauggcacuuguggcuuaguagaaguugaaaaaggcguuuugccucaacuugaacagcccuau<br>guguucaucaaacguucggaugcucgaacugcaccucauggucauguuaugguugagcugguagcag<br>aacucgaaggcauucaguacggucguaguggugagacacuugguguccuugucccucaugugggcga<br>aauaccaguggcuuaccgcaagguucuucuucguaagaacgguaauaaaggagcugguggccauagu<br>uacggcgccgaucuaaagucauuugacuuaggcgacgagcuuggcacugauccuuaugaagauuuuc<br>aagaaaacuggaacacuaaacauagcagugguguuacccgugaacucaugcgugagcuuaacg
Nsp3	uuaugaaagacauucucucucucauuuuguuaacuuagacaaccugagagcuaauaacacuaaaggu<br>ucauugccuauuaauguuauaguuuuugaugguaaaucaaaaugugaagaaucaucugcaaaauca<br>gcgucuguuuacuacagucagcuuaugugucaaccuauacuguuacuagaucaggcauuagugucug<br>auguuggugauagugcggaaguugcaguuaaaauguuugaugcuuacguuaauacguuuucaucaa<br>cuuuuaacguaccaauggaaaaacucaaaacacuaguugcaacugcagaagcugaacuugcaaagaa<br>uguguccuuagacaaugucuuaucuacuuuuauuucagcagcucggcaaggguuuguugauucagau<br>guagaaacuaaagauguuguugaaugucuuaaauugucacaucaaucugacauagaaguuacuggc<br>gauaguuguaauaacuauaugcucaccuauaacaaaguugaaaacaugacaccccgugaccuuggug<br>cuuguauugacuguagugcgcgucauauuaaugcgcagguagcaaaaagucacaacauugcuuugau
Nsp8/9	gccaugcaacguaaguuggaaaagauggcugaucaagcuaugacccaaauguauaaacaggcuagau<br>cugaggacaagagggcaaaaguuacuagugcuaugcagacaaugcuuuucacuaugcuuagaaaguu<br>ggauaaugaugcacucaacaacauuaucaacaaugcaagagaugguuguguucccuugaacauaaua<br>ccucuuacaacagcagccaaacuaaugguugucauaccagacuauaacacauauaaaaauacgugug<br>augguacaacauuuacuuaugcaucagcauugugggaaauccaacagguuguagaugcagauagua<br>aaauuguucaacuuagugaaauuaguauggacaauucaccuaauuuagcauggccucuuauuguaac<br>agcuuuaagggccaauucugcugucaaauuacagaauaaugagcuuaguccuguugcacuacgacag

	augucuugugcugccgguacuacacaaacugcuugcacugaugacaaugcguuagcuuacuacaacacaacaaagggagguagguuuguacuugcacuguuauccgauuuacaggauuugaaaugggcuaga
Nsp10	ggaagccaauauggaucaagaauccuuugguggugcaucguguugucuguacugccguugccacauagaucauccaaauccuaaaggauuuugugacuuaaaagguaaguauguacaaauaccuacaacuugugcuaaugacccugugguuuuacacuuaaaaacacagucuguaccgucugcgguaugugga aagguuauggcuguaguugugaucaacuccgcgaacccaugcuucagucagcugaugcacaaucguuuuuaaacggguuugcgguguaagugcagcccgucuuacaccgugcggcacaggcacuaguacugaugucguauacagggcuuuugacaucuacaaugauaaaguagcugguuuugcuaaauuccuaaaaacuaauuguugucgcuuccaagaaaaggacgaagaugacaauuuaauugauucuuacuuuguaguuaagagacacacuuucucuaacuaccaacaugaagaaacaauuuauaauuuacuuaaggauuguccagcuguugcuaaacaugacuucuuuaaguuuagaauagacggugacaugguaccacauaucacgucaacgucuuacu
SL4a	uuaaaaucugugu ggcugucacucggcugcaugcuuagugcacucacgcaguauaa
SL7	acguggcuuuggagacuccguggaggaggucuuaucagaggcacgu
SL8	gauggcacuuguggcuuaguagaaguugaaaaaggcguuuugccucaacuugaacagcccuauguguucauc
mSL8	gauAgcaAuuguAAcuuaguagUCACGgaaaagUcguuuugccucaacuugaacagcccuauAugAucauc<br>*(mutant bases are capitalized)*
